# Distribution and molecular characterization of rickettsiae in ticks in Harbin area of Northeastern China

**DOI:** 10.1371/journal.pntd.0008342

**Published:** 2020-06-04

**Authors:** Jian-Wei Shao, Xue-Lian Zhang, Wen-Jun Li, Hui-Lan Huang, Jie Yan

**Affiliations:** 1 Department of Medical Microbiology and Parasitology, Zhejiang University School of Medicine, Hangzhou, Zhejiang, P.R. China; 2 Key Laboratory for Preventive Research of Emerging Animal Diseases, College of Life Science and Engineering, Foshan University, Foshan, Guangdong, P.R. China; Hebrew University Hadassah Medical School, ISRAEL

## Abstract

Tick-borne rickettsioses are world-spreading infectious zoonoses. Ticks serve as reservoirs and vectors for *Rickettsia* and play a key role in transmission of rickettsioses. Most of the Chinese rickettsiosis patients are reported from Northeastern China but the distribution of tick and tick-borne *Rickettsia* species in Northeastern China remain poorly studied. In this study, a total of 1,286 ticks were captured from the seven counties of Harbin, an area in Northeastern China, and the tick-borne *Rickettsia* species were identified by PCR and sequencing of *rrs*, *gltA*, *groEL*, *ompA* and *17-kDa* antigen-encoding genes. Of the 5 identified tick species, *Haemaphysalis longicornis* and *Ixodes persulcatus* were the predominant tick species in the livestock and vegetation, respectively. *Rickettsia raoultii* and “*Candidatus* Rickettsia tarasevichiae” were the two detectable *Rickettsia* species in the ticks with a 28.8% positive rate but no rickettsiae were found in ticks of *Haemaphysalis concinna*. *R*. *raoultii* detected in 37.6% of the *Dermacentor nuttalli*, *Dermacentor silvarum* and *H*. *longicornis* ticks while “*Ca*. R. tarasevichiae” was only present in 22.8% of the *I*. *persulcatus* ticks. In particular, the positive rate of both *R*. *raoultii* and “*Ca*. R. tarasevichiae” in ticks from the livestock (40.7%) was significantly higher than that from the vegetation (19.5%). The results indicate that the tick and tick-borne *Rickettsia* species are diverse in different regions of Harbin due to geographic difference and the ticks from livestock may play a more important role in transmission of rickettsioses to human.

## Author summary

Rickettsiosis is a tick-borne infectious disease of global importance. The disease has been prevailing in Northeastern China but the distribution of tick and tick-borne *Rickettsia* species from different areas of Northeastern China remain poorly studied. We collected a total of 1,286 ticks in the seven counties with different geographic environments of Harbin, an area of Northeastern China, and all the ticks were classified as *Dermacentor nuttalli*, *Dermacentor silvarum*, *Haemaphysalis concinna*, *Haemaphysalis longicornis* or *Ixodes persulcatus*. A total of 28.8% of the ticks tested positive for either *Rickettsia raoultii* or “*Candidatus* Rickettsia tarasevichiae”, in which 37.6% of the *D*. *nuttalli*, *D*. *silvarum* and *H*. *longicornis* ticks were positive for *R*. *raoultii* while 22.8% of the *I*. *persulcatus* ticks were positive for “*Ca*. R. tarasevichiae”. The positive rate of both *R*. *raoultii* and “*Ca*. R. tarasevichiae” in ticks from the livestock (40.7%) was significantly higher than that from the vegetation (19.5%). All the data indicate that ticks in the Harbin area have a high infection rate with *Rickettsia* species and domestic animals may have a tick-livestock rickettsial circulation that may play an important role in transmission of rickettsioses.

## Introduction

Rickettsiae are a large group of Gram-negative obligate intracellular prokaryotic microbes that can cause rickettsioses in human and many animals [1]. These microbes are widely distributed throughout the world, and maintained and transmitted by arthropods such as ticks, fleas, mites and lice [2]. Information about many *Rickettsia* species is available in GenBank, in which approximate 20 species are well characterized as human pathogens [1–3]. Particularly in the recent years, novel *Rickettsia* species that cause human diseases have been continuously reported, such as *R*. *monacensis* in Europe and South Korea [4, 5], and *R*. *sibirica* subsp. *sibirica*, *R*. *raoultii*, *R*. subsp. XY99 and “*Candidatus* R. tarasevichiae” in China [6–9]. At present, the genus *Rickettsia* is classified into four groups: the spotted fever group (SFG) that include *R*. *conorii*, *R*. *rickettsii* and *R*. *japonica*, the typhus group (TG) that include *R*. *typhi* and *R*. *prowazekii*, the ancestral group (AG) with species such as *R*. *bellii* and *R*. *canadensis*) and transitional group (TRG) that contains *R*. *felis* and *R*. *akari* [2,3]. Fever, headache, nausea, anorexia, rash and occasional eschar at the tick biting sites are common clinical manifestations of rickettsioses caused by most rickettsiae [10, 11]. Therefore, it is difficult to distinguish diagnosis of rickettsiosis caused by different *Rickettsia* species based on the clinical signs and symptoms of rickettsiosis patients. More specific and accurate laboratory diagnostic methods, for example, PCR and sequencing and genetic analysis, have been widely employed to diagnose human rickettsiosis in clinic [9].

Until now, the four *Rickettsia* species, *R*. *heilongjiangensis*, *R*. *monacensis*, *R*. *raoultii*, and *R*. *sibirica*, have been identified by cultivation methods, while the seven *Rickettsia* species, *R*. *aeschlimannii*, *R*. *conorii*, *R*. *felis*, *R*. *massiliae*, *R*. *slovaca*, “*Ca*. R. tarasevichiae” and *Ca*. R. jingxinensis, have also been confirmed by genetic molecular methods over the past 30 years in mainland of China [12–18]. In addition, according to phylogenic analysis of target gene loci, several potential novel *Rickettsia* species, such as *Ca*. R. hebeiii, *Ca*. R. tibetani, *Ca*. R. gannanii and *R*. subsp. XY99, have been reported in different areas of China according to the phylogenetic analysis of target gene loci [8, 19–21]. Among the rickettsiae, *R*. *heilongjiangensis*, *Ca*. R. hebeiii, *Ca*. R. tibetani, *Ca*. R. gannanii, *R*. subsp. XY99 and *Ca*. R. jingxinensis were first identified in ticks from mainland of China. More importantly, *R*. *heilongjiangensis*, *R*. *raoultii*, *R*. *sibirica*, *R*. subsp. XY99, and “*Ca*. R. tarasevichiae” have been confirmed as the causative agents of human rickettsioses in mainland of China [22].

Ticks act as the most important arthropod vectors in the world for transmission of microbial pathogens to humans [23]. Previous studies revealed the extensive diversity of rickettsiae in different tick species and geographic areas [1–3]. Since 1982, many different species of *Rickettsia* have been identified as pathogens in rickettsiosis patients in mainland of China, especially in the areas of Northeastern China [22]. In addition, in the recent years, many more rickettsiosis patients have been reported in China due to the application of more sensitive and precise laboratory diagnostic methods and most of these patients were from Northeastern China [17, 22].

The Harbin area is located in the southwest of Heilongjiang province, which is the most northeast province of China. In this area, rickettsiosis cases have been frequently reported in the recent years [6, 9, 24, 25]. However, until now, no information about the circulation of *Rickettsia* in ticks of this area has been available. Therefore, in the present study, we investigated the circulation of ticks and tick-borne *Rickettsia* in natural environments from different regions of Harbin area and the risk of rickettsial infections in the local populations were also estimated.

## Materials and methods

### Ethics statement

The collection of ticks from the body surface of cattle, goats and horses in this study was verbally approved by the animal owners and performed in strict accordance with the National Guidelines for Experimental Animal Welfare of China (2006–398).

### Collection and identification of ticks

**Fig 1:**
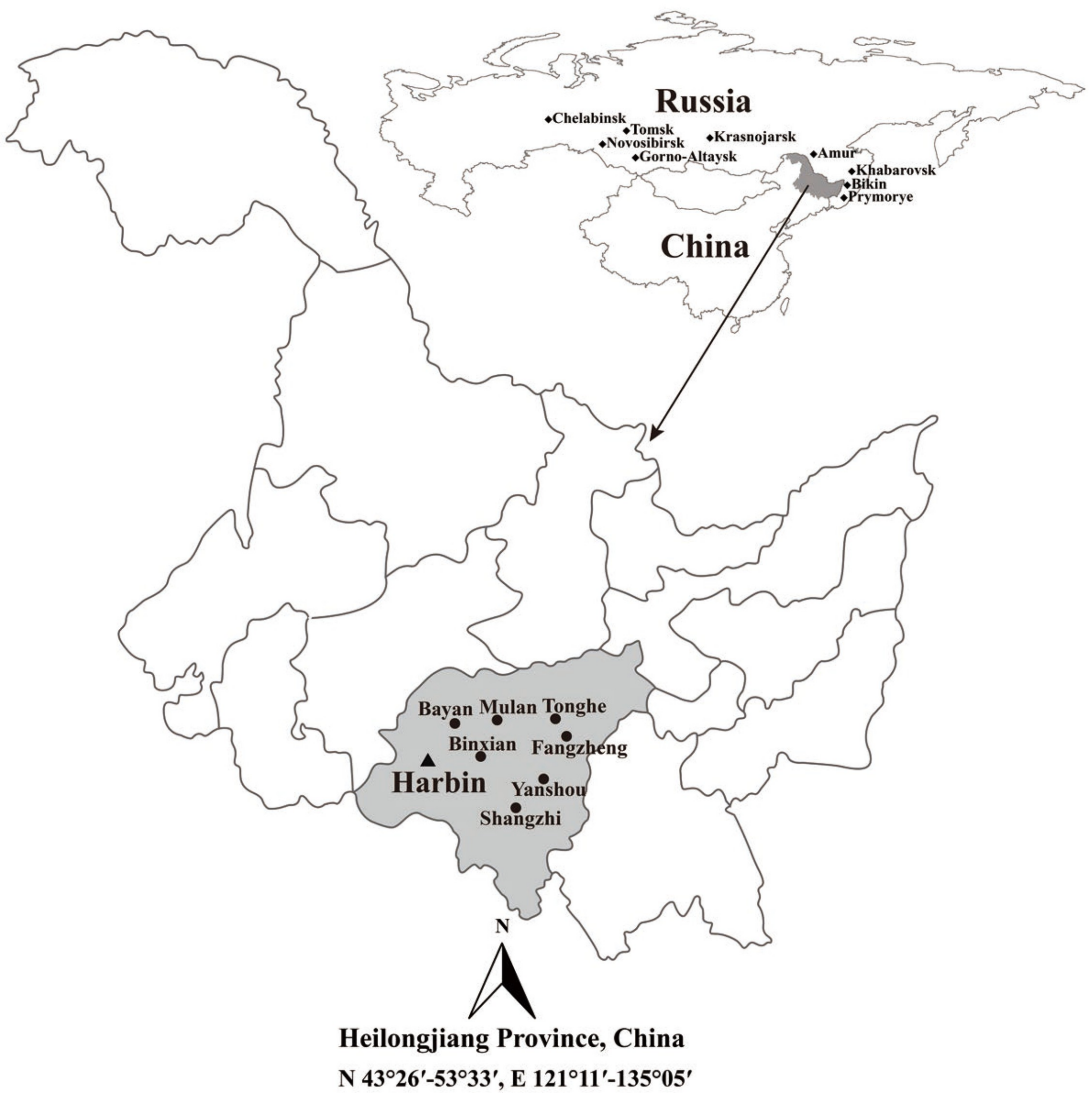
Location of the seven counties for tick sampling in the Harbin area of Heilongjiang province in Northeastern China. This map was plotted by combination of Surfer software version-4 (Golden Software, USA) and Photoshop CS 8.0.1 (Adobe Systems, USA). The black dots indicate the sampling regions in this study. The black diamonds indicate the endemic regions of rickettsioses in Russia.

Adult ticks were captured from the ear, neck, armpit, chest, abdomen and crissum of cattle, goats and horses using tweezers (1–10 ticks per animal), and collected from the different types of vegetative covers by flagging with a white cotton flag (60 cm × 1 m) along its linear transection in the seven counties from the Harbin area of Northeastern China during April to May of 2019 (Fig 1) [26]. The tick species were firstly identified according to their morphology as previously described [27, 28]. Each of the ticks was soaked in 70% ethanol for disinfection and then washed three times with autoclaved double distilled water (ddH_2_O) for homogenization. The total DNAs in each of the homogenized samples were extracted by using an Insect DNA Extraction Kit (D0926, Omega, USA) according to the manufacturer’s protocol and then dissolved in 80 μL TE-buffer in the kit. Using several dilutions of each of the total DNAs as templates, PCR was performed to further identify the ticks using universal primers (Table 1) targeting the 16S ribosomal RNA (*rrs*) genes from different tick species with a High-Fidelity PCR Kit (TaKaRa, China), in which a proof-reading Pfu DNA polymerase was used [29]. In the PCR, a recombinant pUC19 plasmid containing the entire *rrs* gene segment of *D*. *nuttalli*, provided by our laboratory, wild-type pUC19 plasmid and TE-buffer were used as the positive, negative and blank controls, respectively. To prevent cross-contamination, DNA extraction, PCR mixture preparation, amplification and agarose gel electrophoresis were performed in separate rooms, and autoclaved pipettes and filter-containing tips were used. The PCR products were sequenced by Sangon Biotech Co. in China.

**Table 1:** Primers used in this study.

Gene	Primer	Sequence (5’→3’)	Amplicon (bp)	Reference
Tick *rrs*	16SF	F: GTATTTTGACTATACAAAGGTATTG	300	29
	16SR	R: TATTACGCTGTTATCCCTAGAGTATT		
*gltA*	Ric-CS409d	F: CCTATGGCTATTATGCTTGC	720	30
	Ric-CS535d	F: GCAATGTCTTATAAATATTC		
	Ric-CS1258n	R: ATTGCAAAAAGTACAGTGAACA		
	Ric-CS2d	F: ATGACCAATGAAAATAATAAT	1,200	9
	Ric-CSEndr	R: CTTATACTCTCTATGTACA		
*17-kDa*	Rr17k.1p	F: TTTACAAAATTCTAAAAACCAT	450	31
	Rr17k.90p	F: GCTCTTGCAACTTCTATGTT		
	Rr17k.539n	R: TCAATTCACAACTTGCCATT		
Rickettsial *rrs*	Ric-16SF	F: GAACGAACGCTATCGGTATGC	1,390	This study
	Ric-16SR1	R: AATTTTACCGTGGTTGGCTGC		
	Ric-16SR2	R: TGCCTCTTGCGTTAGCTCAC		
*groEL*	Ric-ESL-F1	F: GGTAAATGGGCAGGYACCGAA	1,580	This study
	Ric-ESL-R1	R: GAAGCAACRGAAGCAGCATCTTG		
	Ric-ESL-F2	F: ATCGTTATGAAAGAAAGCGAYG		
	Ric-ESL-R2	R: AGWGCAGTACGCACTACTTTAGC		
*ompA*	Rr190k.71p	F: TGGCGAATATTTCTCCAAAA	530	31
	Rr190k.602n	R: AGTGCAGCATTCGCTCCCCCT		
	Rr190k.720n	R: TGCATTTGTATTACCTATTGT		

F: Forward primer. R: Reverse primer. Y = C or T. W = A or T

### Detection of rickettsiae in ticks

The total DNAs in the homogenized samples of the ticks were extracted as above. Using the DNAs as templates, rickettsial DNAs were detected by nested-PCRs with the primers targeting a 720-bp citrate synthase encoding gene (*gltA*) and a 450-bp 17 kilodalton antigen encoding gene (*17-kDa*) segment as previously described [30, 31]. Subsequently, both the 720-bp *gltA* and *17-kDa* gene segment positive total DNA samples were selected for identification of *Rickettsia* species by PCR with the primers (Table 1) targeting the nearly entire *rrs* gene (1,390 bp), *gltA* gene (1,200 bp) and 60-kDa heat shock protein encoding gene (*groEL*, 1,580 bp), and the partial segment of outer membrane protein A encoding gene (*ompA*, 530 bp) [9, 31]. In particular, the reported primers targeting rickettsial *rrs* gene by PCR and *groEL* genes in semi-nested PCR produced smaller products (813 and 217 bp) [32, 33]. To increase the sensitivity, specificity and efficiency of rickettsial identification, the primers targeting rickettsial *rrs* and *groEL* gene by semi-nested PCR with larger products were designed after analysis of the rickettsial *rrs* and *groEL* gene sequences in GenBank. The PCR products were examined by agarose gel using an Image Analyzer (Bio-Rad, USA) after electrophoresis. The amplified DNA fragments in the gels with expected sizes were extracted using a Gel Extraction Kit (Qiagen, USA) and then cloned into pMD19-T plasmid (TaKaRa) according to the manufacturers’ protocols for sequencing to identify rickettsial species. For PCR detection, the High-Fidelity PCR Kit and prevention of cross-contamination were the same as above. The DNAs from *Rickettsiae japonica* and *Haemaphysalis concinna*, provided by our laboratory, and TE-buffer were used as the positive, negative and blank controls in the PCR, respectively.

### Analysis of sequence homology of the genes from ticks and rickettsiae

The obtained nucleotide sequences from the target gene segments of ticks and rickettsiae were edited and assembled using the SeqMan program (DNASTAR, Madison, WI) and aligned using the Clustal W method in the Lasergene program [34], and then compared with the corresponding sequences in GenBank using BLAST software. The following sequences were used for comparison: MN448327-MN448342 for the *rrs* genes of ticks (300 bp) while MN450395-MN450401 for the *gltA* genes (1,200 bp), MN446743-MN446749 for the *rrs* genes (1,390 bp), MN450402-MN450408 for the *groEL* genes (1,580 bp), and MN450409-MN450415 for the *ompA* genes (530 bp) of rickettsiae.

### Genetic and phylogenic analysis of the ticks and rickettsiae

The best-fit nucleotide substitution models for phylogenetic analysis based on the target genes from the ticks and rickettsiae were determined using jModel Test [35]. Phylogenic trees were constructed using the Maximum likelihood (ML) method in the PhyML v3.0 software [36]. The boot strap support values calculated from 1000 replicates were used to test the reliability of branches in the trees and values over 70% were considered as significant difference for presentation. All phylogenic trees were mid-point rooted for purpose of clarity.

### Statistical data analysis

Statistical analysis of the obtained data was performed using the Statistical Package for Social Sciences Version 21.0 software (SPSS, Chicago, IL, USA). The Chi-square test or Fisher’s exact test was used for calculating the *P* values to determine differences of the positive rates in the ticks and rickettsiae. Statistical significance was defined as *P*<0.05.

## Results

### Species and distribution of the collected ticks

**Fig 2:**
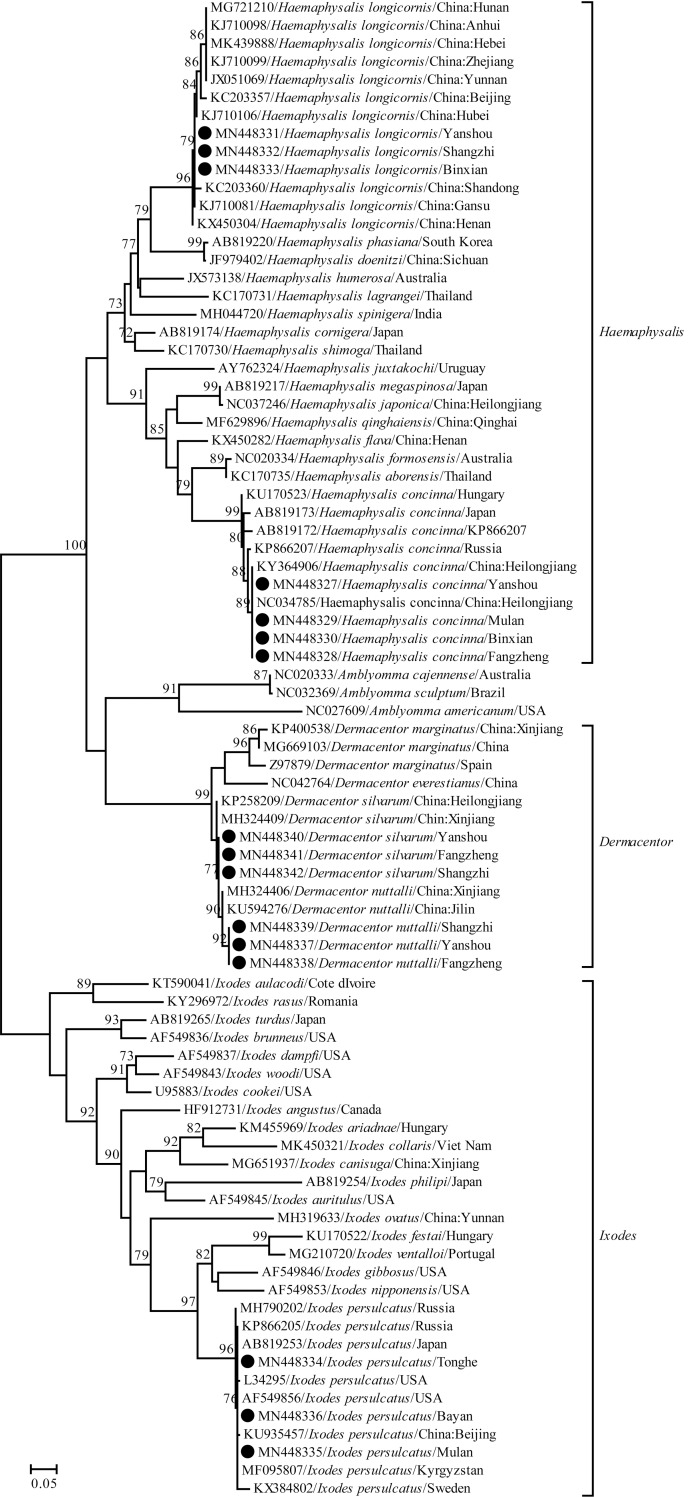
Identification of the ticks based on phylogenic analysis with the *rrs* gene. The black dots indicate the representative sequences of *rrs* gene segments (300 bp) from the ticks in the different sampling regions.

A total of 1,286 adult ticks were collected from the seven sampling regions of the Harbin area and all the ticks were classified as one of five different species belonging to three different genera of ticks according to their morphological characteristics and *rrs* gene sequencing data, namely *Dermacentor nuttalli* (9.6%, 123/1286), *Dermacentor silvarum* (12.8%, 165/1286), *Haemaphysalis concinna* (8.2%, 106/1286), *Haemaphysalis longicornis* (30.6%, 393/1286) and *Ixodes persulcatus* (38.8%, 499/1286) (Fig 1 and Table 2). *H*. *longicornis* was the predominant tick species (63.8%, 393/616) in Yanshou (65.3%, P = 112.05–166.33, *P* < 0.05), Shangzhi (50.8%, P = 13.25 and 53.10, *P* < 0.05) and Binxian (76.7%, P = 71.88, *P* < 0.05) county which have similar geographic environments and similar vegetative covers while *I*. *persulcatus* was the predominant (88.6%, 294/332) or unique tick species in Tonghe (87.6%, P = 181.88, *P* < 0.05) and Mulan (87.6%, P = 213.16, *P* < 0.05) regions or in Bayan region, which also have the same climates and similar geographic environments (Table 2). Among the ticks, 563 were captured from different domestic animals, while 723 were collected from different types of vegetative covers (Table 3). The same five tick species were captured from cattle and goats while three of the five tick species were found on horses, with the absence of *D*. *nuttalli* and *H*. *concinna* (Table 3). *H*. *longicornis* (51.2%, 288/563) was the predominant tick species on cattles (54.8%, P = 64.58–145.87, *P* < 0.05), goats (47.0%, P = 40.83–75.16, *P* < 0.05) and horses (50.0%, P = 9.23 and 18.36, *P* < 0.05), while *I*. *persulcatus* (62.1%, 381/614) was the predominant tick species in forest shrub (67.6%, P = 213.37 and 234.02, *P* < 0.05) and hilly grass/shrub (50.7%, P = 20.84–80.35, *P* < 0.05), but it could not be detected in farm grassland (Table 3). The identification of ticks based on phylogenic analysis with the *rrs* gene segment (300 bp) from the five tick species and sequences from GenBank is shown in Fig 2.

**Table 2:** Ticks collected from different sampling regions.

Region	Coordinate	Geography and climate	Tick (n)	Species / number
Yanshou	N 45°31’	Cold temperate continental monsoon climate, abundant rainfall, low mountain-hilly terrain, broadleaf forest and shrub grassland.	239	*D*. *nuttalli* / 21
	E 128°44’			*D*. *silvarum* / 42
				*H*. *concinna* / 20
				*H*. *longicornis* / 156*
Fangzheng	N 45°50’	The geography and climate are the same as in Yanshou.	133	*D*. *nuttalli* / 36
	E 128°49’			*D*. *silvarum* / 70
				*H*. *concinna* / 27
Shangzhi	N 45°12’	Broadleaf-conifer forest. The others are the same as in Yanshou.	201	*D*. *nuttalli* / 66
	E 128°01’			*D*. *silvarum* / 33
				*H*. *longicornis* / 102*
Binxian	N 45°44’	The geography and climate are the same as in Yanshou.	176	*H*. *concinna* / 41
	E 127°25’			*H*. *longicornis* / 135*
Tonghe	N 45°58’	Mountain terrain. The others are the same as in Shangzhi.	161	*D*. *silvarum* / 20
	E 128°45’			*I*. *persulcatus* / 141*
Mulan	N 45°56’	Mid-temperate continental monsoon climate. The others are the same as in Tonghe.	171	*H*. *concinna* / 18
	E 128°01’			*I*. *persulcatus* / 153*
Bayan	N 46°04’	Plain and low mountain terrain and shrub grassland and conifer forest. The others are the same as in Mulan.	205	*I*. *persulcatus* / 205
	E 127°23’			

*H. *longicornis* and *I*. *persulcatus* were the predominant tick species in the local regions (*P*<0.05).

**Table 3:** Ticks collected from different sources.

Source	Animal/Geomorphy	Tick (n)	Species/number
Livestock	Cattle	261	*D*. *nuttalli* / 16
			*D*. *silvarum* / 21
			*H*. *concinna* / 27
			*H*. *longicornis* / 143*
			*I*. *persulcatus* / 54
	Goat	202	*D*. *nuttalli* / 25
			*D*. *silvarum* / 30
			*H*. *concinna* / 17
			*H*. *longicornis* / 95*
			*I*. *persulcatus* / 35
	Horse	100	*D*. *silvarum* / 21
			*H*. *longicornis* / 50*
			*I*. *persulcatus* / 29
Vegetation	Forest shrub	411	*D*. *silvarum* / 71
			*H*. *concinna* / 62
			*I*. *persulcatus* / 278^#^
	Hilly grass/shrub	203	*D*. *nuttalli* / 20
			*D*. *silvarum* / 22
			*H*. *longicornis* / 58
			*I*. *persulcatus* / 103^#^
	Farm grass	109	*D*. *nuttalli* / 62
			*H*. *longicornis* / 47

* or ^#^the two tick species were significantly more than the other tick species from the livestock and shrub/grasslands, respectively (*P*<0.05).

### Rickettsiae in the collected ticks

Nested-PCR showed that 43.9% (54/123) of the *D*. *nuttalli* ticks, 46.7% (77/165) of the *D*. *silvarum* ticks, 31.8% (125/393) of the *H*. *longicornis*, in the ticks and 22.8% (114/499) in the *I*. *persulcatus* ticks, but not the *H*. *concinna* ticks, were positive for both the *Rickettsia*-specific 720-bp *gltA* and 450-bp *17-kDa* gene segments and the total positive rate of all ticks was 28.8% (370/1286) (Table 4) PCR and sequence analysis of rickettsial *rrs*, *gltA* (1200 bp), *groEL* and *ompA* genes showed that only two species of *Rickettsia*, *R*. *raoultii* in the *D*. *nuttalli*, *D*. *silvarum* and *H*. *longicornis* (69.2%, 256/370) ticks and “*Ca*. *R*. *tarasevichiae*” in the *I*. *persulcatus* ticks (30.8%, 114/370), were identified from the 370 rickettsial *gltA* (720 bp) and *17-kDa* gene positive samples (Table 4). However, the two *Rickettsia* species were not found in the same sample of the *Rickettsia*-positive ticks. DNA of *R*. *raoultii* was detected in the ticks from all the different sources but its positive rate in ticks from the domestic animals (32.0%, 180/563) was significantly higher than that from the vegetative covers (10.5%, 76/723) (P = 91.42, *P*<0.05). In addition, *R*. *raoultii* was the unique *Rickettsia* species detected in ticks from the Yanshou, Fangzheng, Shangzhi and Binxian regions and the “*Ca*. R. tarasevichiae” was only found in the ticks from Mulan and Bayan regions, probably due to their noticeable geographic differences. The total positive rate of both *R*. *raoultii* and “*Ca*. R. tarasevichiae” in ticks from the domestic animals (40.7%, 229/563) was significantly higher than that from the vegetative covers (19.5%, 141/723) (P = 69.24, *P*<0.05).

**Table 4:** *Rickettsia* species in ticks from different sampling regions.

Region	Source		Tick species / n	*Rickettsia* species / infection rate (n / %)	
				*R*. *raoultii*	“*Ca*. R. tarasevichiae”
Yanshou	Livestock	Cattle	*D*. *nuttalli* / 4	3 / 75.0	0
			*D*. *silvarum* / 9	5 / 55.6	0
			*H*. *longicornis* / 59	35 / 59.3	0
		Goat	*D*. *silvarum* / 15	11 / 73.3	0
			*H*. *concinna* / 17	0	0
			*H*. *longicornis* / 41	20 / 48.8	0
	Vegetation	Forest shrub	*D*. *silvarum* / 18	4 / 22.2	0
			*H*. *concinna* / 3	0	0
		Hilly grass/shrub	*H*. *concinna* / 3	0	0
			*H*.*longicornis* / 38	3 / 7.9	0
		Farm grassland	*D*. *nuttalli* / 17	6 / 35.3	0
			*H*.*longicornis* / 18	3 / 16.7	0
Total			239	90 / 37.7	0
Fangzheng	Livestock	Cattle	*D*.*silvarum* / 12	6 / 50.0	0
			*H*.*concinna* / 27	0	0
		Goat	*D*.*silvarum* / 15	8 / 53.3	0
		Horse	*D*.*silvarum* / 21	12 / 57.1	0
	Vegetation	Forest shrub	*D*.*silvarum* / 22	14 / 63.6	0
		Farm grassland	*D*. *nuttalli* / 36	10 / 27.8	0
Total			133	50 / 37.6	0
Shangzhi	Livestock	Goat	*D*. *nuttalli* / 25	16 / 64.0	0
			*H*.*longicornis* / 54	10 / 18.5	0
		Cattle	*D*. *nuttalli* / 12	7 / 58.3	0
			*H*.*longicornis* / 28	5 / 17.9	0
	Vegetation	Forest shrub	*D*. *silvarum* / 31	11 / 35.5	0
		Hilly grass/shrub	*D*. *nuttalli* / 20	9 / 45.0	0
			*D*. *silvarum* / 2	0	0
			*H*.*longicornis* / 20	2 / 10.0	0
		Farm grassland	*D*. *nuttalli* / 9	3 / 33.3	0
Total			201	63 / 31.3	0
Binxian	Livestock	Cattle	*H*.*longicornis* / 56	23 / 41.1	0
		Horse	*H*.*longicornis* / 50	19 / 38.0	0
	Vegetation	Forest shrub	*H*.*concinna* / 41	0	0
		Farm grassland	*H*.*longicornis* / 29	5 / 17.2	0
Total			176	47 / 26.7	0
Tonghe	Livestock	Cattle	*I*.*persulcatus* / 40	0	12 / 30.0
	Vegetation	Forest shrub	*I*. *persulcatus* / 28	0	4 / 14.3
		Hilly grass/shrub	*D*.*silvarum* / 20	6 / 30.0	0
			*I*. *persulcatus* / 73	0	12 / 16.4
Total			161	6 / 3.7	28 / 17.4
Mulan	Livestock	Goat	*I*.*persulcatus* / 17	0	5 / 29.4
		Horse	*I*.*persulcatus* / 29	0	16 / 55.2
	Vegetation	Forest shrub	*H*.*concinna* / 18	0	0
			*I*.*persulcatus* / 77	0	13 / 16.9
		Hilly grass/shrub	*I*.*persulcatus* / 30	0	5 / 16.7
Total			171	0	39 / 22.8
Bayan	Livestock	Cattle	*I*. *persulcatus* / 14	0	8 / 57.1
		Goat	*I*. *persulcatus* / 18	0	8 / 44.4
	Vegetation	Forest shrub	*I*. *persulcatus* / 173	0	31 / 17.9
Total			205	0	47 / 22.9

### Genetic and phylogenic analysis of the identified rickettsiae

**Fig 3:**
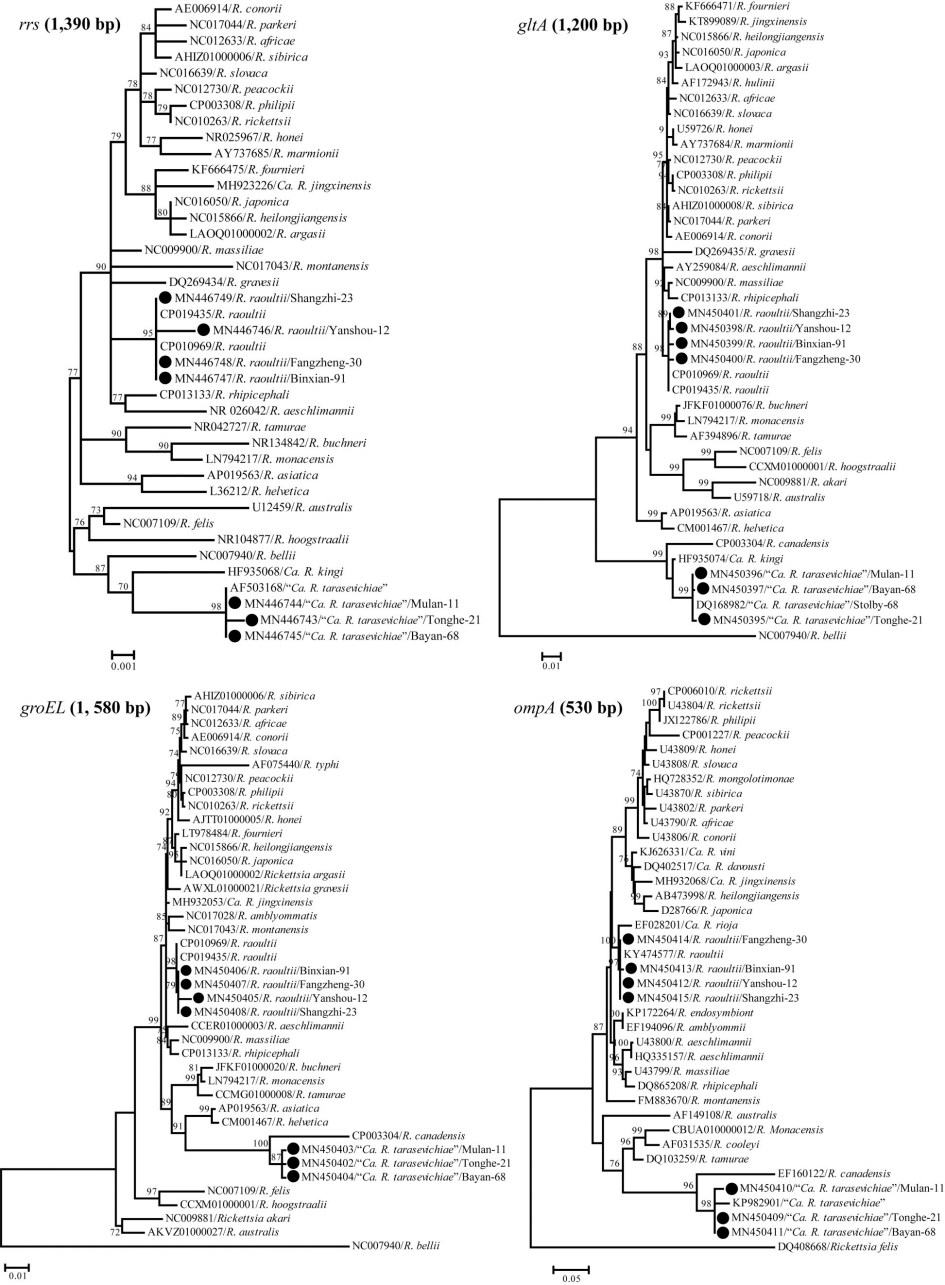
Phylogenic tree based on the *rrs*, *gltA*, *groEL* and *ompA* genes of rickettsiae. The genetic identity among different *Rickettsia* species was inferred by maximum-likelihood method implemented in PhyML v3.0, and rooted by midpoint method. The black dots indicate the nucleotide sequences of *rrs* (1,390 bp), *gltA* (1,200 bp), *groEL* (1,580 bp) and *ompA* (530 bp) gene segments from *R*. *raoultii* and “*Ca*. R. tarasevichiae” in the different sampling regions.

Sequencing data revealed that the *rrs*, *gltA* (1,200 bp), *groEL* and *ompA* gene segments from the 256 strains of *R*. *raoultii* and 114 strains of “*Ca*. R. tarasevichiae” identified in this study presented 99.6%-100% nucleotide sequence identities. The nucleotide sequence identities of the *rrs*, *gltA* (1,200 bp), *groEL* and *ompA* gene segments from the 256 *R*. *raoultii* strains displayed 99.6%-100%, 99.4%-100%, 99.2%-100% and 99.2%-100% nucleotide sequence identity, respectively, compared with the corresponding genes in the whole genome of *R*. *raoultii* strain IM16 (GenBank: CP019435.1), an isolate from a patient in Northern China. For the *rrs*, *gltA* (1,200 bp) and *ompA* gene segments from the 114 “*Ca*. R. tarasevichiae” strains, the nucleotide sequence identities were 99.9%-100%, 99.8%-100% and 98.8%-100%, respectively, compared with the *rrs* and *gltA* genes of a “*Ca*. R. tarasevichiae” strain (GenBank: AF503168.1 and AF503167.2) [37], and the *ompA* gene of “*Ca*. R. tarasevichiae” strain M-R217 (GenBank: KU361217.1), respectively. Since no *groEL* gene sequences of “*Ca*. R. tarasevichiae” could be found in GenBank and a previous study reported that the *rrs* and *gltA* genes of “*Ca*. R. tarasevichiae” had the highest nucleotide sequence identities (98.0% and 96.0%) with those of *Rickettsia canadensis* among different *Rickettsia* species [37], the nucleotide sequence identities of *groEL* gene segments from the 114 “*Ca*. R. tarasevichiae” strains were compared with the *groEL* gene of *R*. *canadensis* strain McKiel (GenBank: CP000409.1) and the sequence identities were 96.8%-97.0%. The phylogenetic tree based on comparison of the four rickettsial genes with those from GenBank is shown in Fig 3.

## Discussion

Ticks act as the main reservoir hosts of many microbial pathogens as well as the major transmission vector of the pathogens to both human and animals in tropical and subtropical areas [23, 38]. *Rickettsia* is a large group of heterogeneous obligate intracellular microbes and ticks serve as the major host and vector for most *Rickettsia* species [1–3]. Since many *Rickettsia* species can cause different types of human and animal rickettsioses and the geographic distribution of tick species and tick-borne *Rickettsia* species are considerably various, investigation of tick-borne rickettsiae in different areas is significant for prevention and control of rickettsioses in different areas.

In Northeastern China, at least eighteen species belonging to seven genera of ticks have been reported due to the profuse and manifold vegetative covers serving as habitats for ticks [39]. Among the ticks, *I*. *persulcatus*, *H*. *longicornis* and *D*. *silvarum* are the most predominant tick species in the area [17, 39]. In the present study, five tick species, *D*. *nuttalli*, *D*. *silvarum*, *H*. *concinna*, *H*. *longicornis* and *I*. *persulcatus*, could be found in the seven counties/regions of the Harbin area, but the number of tick species from these sampling regions presented a notable difference. For example, *I*. *persulcatus* was the unique tick species found in the Bayan region. *H*. *longicornis* was the predominant tick species in Yanshou, Shangzhi and Binxian regions (63.8%) while *I*. *persulcatus* was the predominant tick species in Tonghe and Mulan regions (88.6%), probably due to their distinct differences in climates, geographic environments and vegetative covers. On the other hand, *H*. *longicornis* was significantly more associated with the domestic animals (51.2%, 288/563) than with the different types of vegetative covers (33.7%, 105/312) (*P*<0.05), and it could not even be found from the forest shrub. In contrast, *I*. *persulcatus* was significantly less associated with the domestic animals (21.0%, 118/563) than with the forest shrub and hilly grass/shrub (62.1%, 381/614) (*P*<0.05). Previous reports showed that *H*. *longicornis* prefers to parasitize on artiodactyl/perissodactyl mammalian herbivores, such as cattle, goats and horses, while adult *I*. *persulcatus* parasites on multiple wild and domestic mammalian animals and several kinds of birds [40, 41]. During the free-living stage, *H*. *longicornis* likes to conceal in ground grasses, while *I*. *persulcatus* is encountered in forests of taiga in the mountains. The distribution of *I*. *persulcatus* and *H*. *longicornis* found in this study was corresponding with previous reports. These data indicate that *H*. *longicornis* and *I*. *persulcatus* are the predominant tick species in the Harbin area, while the different geographic environments, vegetative covers, climates and sampling sources can account for the diversity in distribution of different tick species.

Although a total of 1286 ticks belonging to five tick species were collected in this study, only two *Rickettsia* species (*R*. *raoultii* and “*Ca*. R. tarasevichiae”) could be found. Previous studies reported that *H*. *concinna* is an important vector of rickettsiae such as *R*. *raoultii*, “*Ca*. R. tarasevichiae”, *R*. *heilongjangensis* and *R*. *hulinii* [17, 42, 43]. However, no rickettsiae were detected in any of the *H*. *concinna* ticks collected in this study. *R*. *raoultii* was firstly detected in *D*. *nuttalli* and *R*. *pumilio* ticks in 1999 in the former Soviet Union [44]. Subsequently, *R*. *raoultii* was found in 12 species belonging to 6 genera of ticks in Europe, North Africa and Asia and the tick species belonging to the genus *Dermacentor* have been confirmed as the major reservoir and vector of this rickettsial species [38, 45–51]. *R*. *raoultii* is widely distributed in Northeastern China and the Far-East/Siberia areas of Russia and ticks belonging to *Dermacentor* species were confirmed as the common natural hosts [49–54]. In this study, *R*. *raoultii* was detected in *D*. *nuttalli*, *D*. *silvarum*, and *H*. *longicornis* ticks in Harbin, an area of Northeastern China. “*Ca*. R. tarasevichiae” is an emerging tick-borne *Rickettsia* species initially found in *I*. *persulcatus* ticks in Russia [37]. In this study, “*Ca*. R. tarasevichiae” was also solely found in *I*. *persulcatus* ticks. The tick and tick-borne *Rickettsia* species found in this study were similar to those reported from Russia, probably due to the adjacency of Northeastern China and the Far-East/Siberia areas of Russia, with similar natural environments and habitats for ticks. Several *rrs* and *groEL* gene segments of *R*. *raoultii* strains and *rrs* gene segments of “*Ca*. R. tarasevichiae” strains were different from the others in tree branch lengths of the phylogenetic tree, probably due to single nucleotide polymorphisms (SNPs) in the genes from different strains [55, 56]. All the data indicate that the different geographic environments act as the major influenting factor for distribution of tick and tick-borne *Rickettsia* species.

Both *R*. *raoultii* and “*Ca*. R. tarasevichiae” have been confirmed as causative agents of human rickettsioses. Most of rickettsiosis patients have a common pathological change of blood vessel endothelial injury at early stage during infection [57]. *R*. *raoultii* can cause human disease called tick-borne lymphadenopathy (TIBOLA) with the clinical features necrotic erythema, eschar and cervical adenopathies [45, 58]. The initial TIBOLA Chinese cases were reported in Northeastern China in 2014 [24]. “*Ca*. R. tarasevichiae” is a member of rickettsiae in the spotted fever group and clinical signs and symptoms of patients infected by this pathogen are fever, headache, nausea, eschar and lymphadenopathy [9]. The “*Ca*. R. tarasevichiae”-infected Chinese patients were also initially found in Northeastern China in 2013 [59]. In the past years, nearly all of the emerging or re-emerging tick-borne human rickettsioses have been found in Northeastern China including Heilongjiang province [6, 9, 24, 25]. In this study, approximately 30% of the collected ticks from the Harbin area, which is located in the southwest of Heilongjiang province, were found to carry either *R*. *raoultii* or “*Ca*. R. tarasevichiae”. In particular, the total positive rate of both *R*. *raoultii* and *Ca*. R. tarasevichiae in ticks from the domestic animals (40.7%) was significantly higher than that from the different types of vegetative covers (19.5%). Except for the preference of host and habitat, co-feeding of domestic animals is also a risk factor that increases tick-borne rickettsial infections among the animals. These data indicate that the circulation of rickettsial infections in the domestic animals in the Harbin area of Northeastern China is an important subject for investigation and may play an important role in prevention and control of transmission of tick-borne rickettsioses in local populations.

Flagging is a typical method for collection of ticks from vegetation, but it is unable to capture all the given groups of ticks in the sampling sites due to many influening factors, such as different types of vegetation, behavior and habitat characteristics of different tick species, and climate [60]. The capture of ticks by tweezers from the livestock is also influenced by the preferred infestation positions on the different animals and developmental stages of the ticks. However, the large-sample of 1286 ticks collected in this study should still reflect the general distribution and predominant species of ticks in the different geographic regions of the Harbin area. Taken together, this study revealed the predominant tick species (*H*. *longicornis* and *I*. *persulcatus*) and tick-borne *Rickettsia* species (*R*. *raoultii* and “*Ca*. R. tarasevichiae”) in the Harbin area of Northeastern China, as well as the more important role of domestic animals in transmission of rickettsioses, as reflected by the higher positive rates of *Rickettsia*-infected ticks.
